# Differential Associations of Executive-Function Components with Initial Status and Change in Chinese Character Reading During Preschool

**DOI:** 10.3390/jintelligence14100266

**Published:** 2026-10-16

**Authors:** Yanyan Ye, Yiwei Zhao, Xiujie Yang

**Affiliations:** 1 Bay Area School of Applied Psychological Sciences, Beijing Normal University at Zhuhai, Zhuhai 519087, China; yanyanye@bnu.edu.cn; 2 Beijing Key Laboratory of Applied Experimental Psychology, National Demonstration Center for Experimental Psychology Education, Faculty of Psychology, Beijing Normal University, Beijing 100875, China; 202221061028@mail.bnu.edu.cn; 3 Institute of Developmental Psychology, Faculty of Psychology, Beijing Normal University, Beijing 100875, China

**Keywords:** executive function, Chinese character reading, working memory, cognitive flexibility, kindergarten

## Abstract

Executive functions may contribute differently to early literacy, but it remains unclear whether their component-specific associations differ with respect to children’s existing reading knowledge and subsequent growth. This question is particularly relevant in character-based writing systems, where learning to read requires children to establish and retrieve associations between visually complex written forms and their pronunciations. This one-year longitudinal study followed 131 kindergarten children across three timepoints and examined whether baseline working memory, inhibitory control, and cognitive-flexibility task performance was associated with initial status and subsequent change in Chinese character reading. A latent growth model was estimated with age, gender, nonverbal intelligence, and socioeconomic status as covariates. Verbal working-memory performance was consistently and positively associated with initial reading status but not with subsequent change. No statistically significant unique associations were found for inhibitory-control performance. Cognitive-flexibility performance was not consistently associated with initial status but was negatively associated with subsequent reading change; exploratory analyses suggested that this association was stronger among younger kindergarten children. Overall, the findings indicate that different executive-function components may relate differently to existing reading knowledge and subsequent reading growth during the preschool period. They also highlight the importance of considering developmental stage, task characteristics, and measurement constraints when examining executive function and reading relations across writing systems.

## 1. Introduction

Early literacy development is an important aspect of school readiness and provides a foundation for later academic learning. Before formal schooling begins, children vary considerably in their knowledge of print and written symbols. These early differences are associated with later academic achievement and may remain relatively stable across development (Duncan et al., 2007; Józsa et al., 2022; Ritchie & Bates, 2013). Understanding the cognitive processes associated with such variation may therefore clarify how early reading skills develop during preschool.

Early reading development reflects the contribution of both language-specific skills and broader cognitive processes. In Chinese, phonological, morphological, and orthographic skills support children’s acquisition of written language (Liu et al., 2022; D. J. Pan et al., 2021; L. Wang et al., 2024; Z. Wang et al., 2022). Domain-general cognitive abilities may also contribute to reading development by helping children maintain relevant information, regulate attention, and adapt their behavior to changing demands. Executive function is one such set of abilities and is commonly described in terms of working memory, inhibitory control, and cognitive flexibility (Cartwright & Palian, 2024). These related but distinguishable processes develop substantially during early childhood and have been associated with academic outcomes, including reading (Ahmed et al., 2019; Fuhs et al., 2015; Ribner, 2020; Spiegel et al., 2021; Zhang et al., 2025).

However, evidence concerning executive function and early reading remains mixed and incomplete. Some studies have reported positive associations between executive function and early literacy development (e.g., Morgan et al., 2019; Nguyen & Duncan, 2019), while others suggest that these associations may be modest and or vary depending on the executive-function components, developmental period, and reading outcome examined (e.g., Jacob & Parkinson, 2015; Haft et al., 2019; Karr et al., 2018; Malone et al., 2022; Willoughby et al., 2019). Much of the existing evidence is also cross-sectional, making it difficult to distinguish associations with children’s existing reading knowledge from associations with subsequent development. In addition, studies have often represented executive function using broad composite measures or have focused on only selected components, potentially obscuring differences in the relations of working memory, inhibitory control, and cognitive flexibility with reading.

These questions are particularly relevant during the preschool years, when executive function and early literacy skills are both developing rapidly. They may also be informative in Chinese, where learning to read involves acquiring visually complex written characters and their conventional pronunciations (Chang et al., 2018). Therefore, a longitudinal and component-specific approach can help clarify whether performance on different domain-general executive-function tasks is associated with children’s existing character-reading knowledge, subsequent change in character reading, or both. The present study addressed these questions by examining working memory, inhibitory control, and cognitive flexibility in relation to the development of single-character reading across one year in Chinese kindergarten children.

### 1.1. Early Chinese Character Reading and Its Cognitive Demands

Chinese children may begin to acquire basic knowledge of written characters before entering primary school through everyday routines and informal literacy activities (Ye & McBride, 2022). Therefore, early character knowledge can be viewed as one focused aspect of literacy-related school readiness and a foundation for subsequent reading development (Lei et al., 2011; Lin et al., 2019; McBride-Chang & Ho, 2005).

In the present study, character reading refers specifically to the ability to correctly name isolated single characters. This skill should be distinguished from multi-character word reading. Although the two skills are closely related, the pronunciation of a character may be supported by the lexical context provided by the other characters in a word (Y. Wang & McBride, 2016; D. J. Pan et al., 2021). For example, Chinese kindergarten children read the same characters more accurately when they appeared within familiar multi-character words than when they were presented in isolation, and character reading and word reading showed partly distinct cognitive correlates (Y. Wang & McBride, 2016). The construct examined here is also narrower than reading fluency and reading comprehension. The task assessed whether children could produce the conventional pronunciation of an isolated character; it did not assess reading speed or require children to demonstrate understanding of the character’s meaning.

The Lexical Constituency Model (Perfetti et al., 2005) provides a useful framework for characterizing the processes involved in this task. In this model, lexical representations involve interconnected orthographic, phonological, and semantic information, and research on Chinese character identification indicates that these sources of information become activated over the course of lexical processing (Perfetti & Tan, 1998). For the character-naming task used in the present study, successful performance directly requires children to identify a written character and retrieve its corresponding pronunciation. Semantic information may also be activated during this process, but semantic access is not directly required or assessed. Thus, the present measure is best understood as indexing the accuracy of character-level lexical access, particularly the acquisition and retrieval of associations between orthographic forms and their conventional pronunciations.

The Chinese writing system presents a distinctive demand for acquiring these character representations. Chinese characters consist of strokes and components arranged within two-dimensional configurations. Many characters are semantic–phonetic compounds containing a component that provides information about meaning and another that provides information about pronunciation (Shu et al., 2003). However, these cues are not fully reliable. Children must learn both individual character forms and pronunciations while gradually developing sensitivity to recurring orthographic and phonological patterns within the writing system (McBride, 2016).

Research with Chinese-speaking children supports the importance of multiple language- and script-related skills in character acquisition (Anderson et al., 2013; Li et al., 2012; Lin et al., 2019; Shu & Anderson, 1997; Shu et al., 2000). Domain-general cognitive processes may provide additional support while character representations and form–pronunciation associations are being established. For example, early character learning may require children to temporarily maintain unfamiliar information, manage interference from competing representations, and coordinate different sources of orthographic and phonological information across characters. These demands provide a basis for examining whether components of executive function are associated with children’s existing character knowledge and with subsequent change in character reading. Importantly, such executive processes are not assumed to operate independently of language- and script-specific skills; rather, they may support the learning and use of the representations on which character reading depends.

### 1.2. EF Components and Their Associations with Initial Character-Reading Status and Subsequent Change

Executive function refers to the cognitive processes that support goal-directed behavior, particularly when habitual or automatic responses are insufficient. It is commonly conceptualized as comprising several related but distinguishable components, including working memory, inhibitory control, and cognitive flexibility (Diamond, 2013). Working memory supports the temporary maintenance and manipulation of information; inhibitory control enables the suppression of dominant responses or distracting information; and cognitive flexibility supports adjustment to changing rules, demands, or perspectives. Although these components can be distinguished conceptually (Friedman & Miyake, 2017; Miyake et al., 2000), their differentiation is still developing during early childhood (Garon et al., 2008). Studies of young children often find considerable shared variance among executive-function tasks (Karr et al., 2018; Wiebe et al., 2008). However, examining performance on these components separately may be informative when considering their relations with early reading.

The present study focuses specifically on domain-general executive function, assessed using tasks that do not involve reading. This focus should be distinguished from reading-specific executive processes, in which control is exercised over orthographic, phonological, or semantic information during reading itself (Cartwright, 2012; Cartwright & Palian, 2024). Reading-specific forms of flexibility may be more directly involved in coordinating these sources of information, whereas domain-general executive function reflects broader capacities that operate across different activities. The present study examined the latter to determine whether broader cognitive control abilities assessed outside the reading context are associated with children’s existing character-reading knowledge and subsequent change. These domain-general processes are not assumed to replace or operate independently of language- and script-specific skills. Rather, they may support the acquisition, maintenance, and use of the representations on which character reading depends.

An important distinction is whether EF task performance is associated with children’s existing reading knowledge or with subsequent change in reading. In the present study, initial status represents children’s estimated level of character reading at T1 and thus reflects the character knowledge they had accumulated by that time. Subsequent change reflects change in single-character reading over the following year. Because EF and character reading were both assessed at T1, associations between baseline EF task performance and initial status are concurrent and do not establish temporal direction. In contrast, associations between baseline EF and subsequent change are prospective. Distinguishing these two aspects of reading makes it possible to examine whether an EF component is associated with children’s accumulated character knowledge, their subsequent improvement, or both.

Working memory may be particularly relevant during the early acquisition of written characters. Children may need to temporarily maintain an unfamiliar visual form and its pronunciation, connect this information with existing lexical knowledge, and retrieve the association during later encounters. Working memory may therefore facilitate the establishment and strengthening of associations between character forms and pronunciations across repeated learning experiences. More broadly, meta-analytic evidence indicates a moderate association between working memory and reading across studies and reading outcomes (Peng et al., 2018). Working memory has also been associated with early Chinese reading, although its contribution varies across samples and measures (Fong & Ho, 2022; J. Pan & Lin, 2020; D. J. Pan & Lin, 2023). In Hong Kong kindergarten children, working memory and inhibitory control jointly accounted for unique variance in Chinese word reading after age, vocabulary, and metalinguistic skills were controlled (K. K. Chung & McBride-Chang, 2011). Working memory has also been examined alongside metalinguistic knowledge in relation to character reading among Mandarin-speaking kindergarten children (Yang et al., 2019).

Longitudinal findings, however, suggest that its association with reading may differ depending on whether reading level or subsequent development is considered. Fung et al. (2020) found that working memory was the strongest concurrent EF correlate of Chinese word reading, whereas there was limited evidence that earlier EF predicted subsequent word reading after prior reading performance was taken into account. Ren et al. (2022) similarly found that preschool working memory was associated with Chinese word reading level, whereas an overall behavioral EF measure and parent-rated inhibition and cognitive flexibility predicted growth in word reading from preschool to Grade 3. Taken together, these findings provide a basis for expecting working-memory task performance to be positively associated with initial character-reading status, whereas evidence for its association with subsequent change during preschool remains less certain.

Inhibitory control may support character reading when recognition activates competing information that must be resolved. Visually similar characters may activate competing orthographic representations, and characters or components associated with different pronunciations may create competition at the phonological level. In such situations, inhibitory control may help children maintain the relevant response while suppressing competing or inappropriate alternatives (Fung et al., 2020). Thus, even isolated character naming may involve some degree of competition among lexical candidates. However, the extent to which successful character naming depends on domain-general inhibitory control is likely to vary with children’s knowledge and with the demands of the reading task.

Empirical findings for inhibitory control are correspondingly mixed. Some studies have reported unique or combined contributions of inhibitory control and working memory to Chinese word reading, whereas others have found weak or inconsistent longitudinal associations after accounting for earlier reading and other cognitive or language skills (K. K. Chung & McBride-Chang, 2011; Fung et al., 2020; Yang et al., 2019). Studies using composite EF measures also cannot determine whether an observed association is specifically attributable to inhibitory control (e.g., K. K. H. Chung et al., 2018). Thus, there are theoretical reasons to expect inhibitory control to be relevant when competing representations are activated, but existing evidence does not provide a strong basis for predicting whether inhibitory-control task performance should be associated with either initial character-reading status or subsequent change.

Cognitive flexibility refers to the ability to adjust attention and behavior in response to changing demands or rules (Collette & Van der Linden, 2002). In literacy learning, flexibility may support adjustment to different characteristics of written material and changes in the information that is most useful for identifying or learning a written form. In Chinese character learning, for example, children gradually become sensitive to both the overall configuration of a character and recurring information provided by its components. Domain-general cognitive flexibility may support adjustment of attention across such changing task demands, although it should be distinguished from reading-specific flexibility in coordinating orthographic, phonological, and semantic information during reading itself (Cartwright, 2012; Cartwright & Palian, 2024).

Evidence linking cognitive flexibility to reading has generally been positive, although findings vary across age groups, measures of flexibility, and reading outcomes. Cognitive flexibility has been associated with early reading skills in alphabetic languages (Colé et al., 2014; Tal & Shaul, 2024), and studies of Chinese children have also reported associations between cognitive flexibility or broader executive-function measures and reading outcomes (Fong & Ho, 2022; D. J. Pan & Lin, 2023; Ren et al., 2022). However, direct longitudinal evidence concerning cognitive flexibility and the development of single-character reading during preschool remains limited. Some longitudinal research has linked broader or parent-rated flexibility measures to later reading outcomes or growth, but differences in measurement, age range, and reading tasks make the findings difficult to generalize to behavioral cognitive-flexibility performance and one-year growth in isolated-character reading (Fong & Ho, 2022; Fung et al., 2020; Ren et al., 2022). Therefore, we examined whether baseline cognitive-flexibility task performance was associated with both initial character-reading status and subsequent change. Previous findings suggest a generally positive relation between cognitive flexibility and reading but provide insufficient evidence to determine whether this association should be more evident for existing character knowledge or for subsequent change.

The interpretation of cognitive flexibility may also be particularly sensitive to developmental level during the preschool years. Tasks commonly used to assess cognitive flexibility in young children require not only switching between rules but also maintaining the currently relevant rule and resolving interference from a previously relevant rule (Davidson et al., 2006; Blakey et al., 2016). These control processes develop rapidly during early childhood. Consequently, performance on a domain-general switching task may reflect somewhat different combinations of rule maintenance, conflict resolution, and switching ability at different ages. This developmental complexity provides an additional reason not to assume that cognitive flexibility will show a uniform association with early character reading across the preschool period.

More broadly, the preschool years involve substantial changes in how children engage cognitive control. The Dual Mechanisms of Control framework distinguishes between reactive control, in which control is recruited in response to an immediate demand, and proactive control, in which goal-relevant information is actively maintained in anticipation of upcoming demands (Braver, 2012). Developmental research suggests that proactive forms of control become increasingly available across childhood, whereas younger children rely more heavily on reactive forms of control. This framework does not imply that children at different kindergarten levels can be classified as using one control mode or the other, nor were these control modes directly measured in the present study. It nevertheless provides a developmental basis for expecting associations between domain-general executive function and character reading to vary across the preschool period. Previous research has rarely tested whether relations between particular executive-function components and reading differ across kindergarten levels, leaving this possibility largely unresolved.

### 1.3. The Present Study

Despite increasing interest in the relation between executive function and reading, several limitations remain. Much of the broader evidence has been obtained from children learning alphabetic writing systems and has focused on school-aged children or relatively broad reading outcomes, particularly reading comprehension (D. J. Pan & Lin, 2023; Yang et al., 2019; Yang & Qiao, 2021). Research specifically examining executive function and early Chinese character reading is more limited. Within this literature, many studies have used cross-sectional or concurrent designs, making it difficult to distinguish associations with children’s existing reading knowledge from associations with subsequent development (K. K. H. Chung et al., 2018; Yang et al., 2019).

Longitudinal studies have begun to address this limitation, but they remain relatively few and differ substantially in their age ranges, reading measures, and assessment of executive function. For example, Fung et al. (2020) examined reciprocal relations between executive-function performance and Chinese word reading across two waves among Hong Kong kindergarten children, whereas Liu et al. (2019) followed Hong Kong children during the transition from kindergarten to primary school. Ren et al. (2022) modeled Chinese word reading trajectories from preschool through Grade 3 using a combination of behavioral tasks and parent reports of executive function. These studies provide valuable evidence for longitudinal associations between executive function and Chinese reading, but less is known about how particular domain-general executive-function components are related separately to children’s initial level of isolated single-character reading and to subsequent change during the preschool years.

The present study addressed this gap by following K1 and K2 children in mainland China across three measurement occasions over one year. Character reading was assessed using a single-character-naming task, and working memory, inhibitory control, and cognitive flexibility were assessed using separate behavioral tasks. Latent growth modeling was used to distinguish children’s initial character-reading status from subsequent change. In addition, given the rapid development of cognitive control across the preschool years, we examined whether the associations between executive-function task performance and character reading differed between K1 and K2 children.

This study addressed the following questions: First, what pattern of change characterized single-character reading across the three measurement occasions? Second, were baseline working memory, inhibitory control, and cognitive-flexibility task scores associated with initial character-reading status and subsequent change? Third, did these associations differ between K1 and K2 children?

We expected single-character reading to increase across the study period. Based on the relatively consistent evidence linking working memory to children’s existing reading knowledge, we also expected working-memory task performance to be positively associated with initial character-reading status. Evidence regarding working memory and subsequent reading change is less consistent, and evidence for inhibitory control and cognitive flexibility is mixed or limited, particularly for longitudinal change in single-character reading during preschool. These associations were examined exploratorily. Comparisons between K1 and K2 were theoretically motivated by developmental changes in cognitive control across the preschool period but were also treated as exploratory because existing evidence does not provide a sufficient basis for directional predictions regarding which executive-function components should show stronger associations at either kindergarten level.

## 2. Materials and Methods

### 2.1. Participants

The participants were 131 children attending one public kindergarten in Ma’anshan, Anhui Province, mainland China. The sample included 44 children in K1 and 87 children in K2; 62 were boys. At the first assessment, children had a mean age of 4.98 years (SD = 0.62, range = 3.66–5.81 years).

Children were recruited from all K1 and K2 classrooms in the participating kindergarten. Parents or legal guardians provided written informed consent before data collection, and children provided verbal assent before each assessment. According to kindergarten records, none of the participating children had a diagnosed intellectual disability, uncorrected sensory impairment, or neurological, attention, emotional, or physical condition that would prevent valid participation in the assessments. This study was approved by the University and was conducted in accordance with relevant institutional and ethical guidelines.

The sample size was determined by the number of children enrolled in the participating classrooms whose parents provided consent. Because this study was conducted in a single kindergarten, the sample represents one specific educational and regional context and may not capture variation in instructional practices, literacy exposure, or family background across kindergartens and regions. This limits the generalizability of the findings beyond similar preschool settings. In addition, the K1 and K2 groups were unequal in size, with substantially fewer children in K1. This imbalance reduces the precision and statistical power of comparisons involving kindergarten level, particularly estimates for the smaller K1 group. Accordingly, analyses examining differences between K1 and K2 were treated as exploratory and are interpreted cautiously.

The participating kindergarten used Mandarin as the principal language of instruction and simplified Chinese characters in its educational materials. At the time of this study, the children had not yet entered primary school and had not received systematic, curriculum-based instruction in character reading comparable to that provided in formal primary education. According to the kindergarten curriculum, classroom activities included shared book reading, oral-language activities, environmental-print exposure, or other relevant activities, but did not include regular direct instruction or formal testing of character reading. Children may nevertheless have encountered written characters through picture books, environmental print, digital media, and informal interactions at home or in kindergarten. The amount and nature of this informal literacy exposure were not assessed in the present study.

### 2.2. Measures

#### 2.2.1. Character Reading

Character reading was assessed using the character-reading subtest of a standardized Chinese reading measure (Yang & McBride, 2020). Children were shown 27 printed Chinese characters individually and asked to read each character aloud. The items included both single-component and compound characters: 21 were single-component characters and 6 were compound characters composed of multiple components or radicals. A response was scored as correct when the child produced the conventional pronunciation of the character. Correct responses received one point, and incorrect or omitted responses received zero points, yielding a possible total score of 0–27. Higher scores indicated that the child could correctly name a larger number of isolated characters.

The task assessed the accuracy of retrieving the pronunciation of visually presented individual characters. The same task was administered at all three assessment occasions. Internal consistency was high at T1, T2, and T3, with Cronbach’s α values of 0.97, 0.96, and 0.96, respectively. In the present sample, the measure showed increasing upper-bound compression across measurement occasions, particularly at T3. Thus, although the task captured individual differences in character-reading accuracy, its sensitivity to differences among higher-performing children decreased over time; this issue was considered in the sensitivity analyses and interpretation of the growth estimates.

#### 2.2.2. Working Memory

Working memory was assessed at T1 using a backward digit-span task (Chen et al., 2022). The examiner read sequences of digits aloud, and the child was asked to repeat each sequence in reverse order. The task included seven levels of increasing difficulty, with two trials at each level. Administration was discontinued after the child responded incorrectly to both trials at the same level. The score was the total number of digit strings correctly recalled in reverse order, with higher scores indicating better task performance. Internal consistency in the present sample was α = 0.87.

#### 2.2.3. Inhibitory Control

Inhibitory control was assessed at T1 using the Grass–Snow task (Chen et al., 2022). Two cards, one depicting green grass and the other depicting white snow, were placed in front of the child. Children were instructed to point to the card opposite to the examiner’s spoken cue: they were required to point to the snow card when the examiner said “grass” and to the grass card when the examiner said “snow”.

After four practice trials, children completed 16 test trials. Each correct response received one point, yielding a possible score of 0–16. Higher scores indicated better performance in suppressing the prepotent response and applying the opposite-response rule. Internal consistency was α = 0.84. In the present sample, scores were strongly concentrated near the upper limit of the task, indicating restricted variability and a substantial ceiling effect. This range restriction reduced the task’s sensitivity to individual differences in inhibitory control and may have limited its ability to detect associations with character reading.

#### 2.2.4. Cognitive Flexibility

Cognitive flexibility was assessed at T1 using the Dimensional Change Card Sort task (DCCS; Chen et al., 2022). Children first sorted cards according to one dimension, color, and were then instructed to switch and sort the cards according to a second dimension, shape. In the final mixed phase, the required sorting rule depended on whether the card had a border: bordered cards were sorted by shape, whereas unbordered cards were sorted by color.

Each correct sort received one point. The scoring procedure yielded a maximum possible score of 17, with higher scores indicating better performance in switching and coordinating sorting rules. Internal consistency in the present sample was α = 0.61. Thus, although the task provided a behavioral indicator of cognitive flexibility, the score should be regarded as an imperfect measure of the broader construct, and associations involving this task were interpreted cautiously.

#### 2.2.5. Nonverbal Intelligence

Nonverbal intelligence was assessed at T1 using a short form of Raven’s reasoning test (Yang & Meng, 2016). The measure was administered through an online assessment platform under examiner supervision. Children selected the response that correctly completed each visual pattern. Higher scores indicated stronger nonverbal reasoning performance. Internal consistency was α = 0.76.

#### 2.2.6. Socioeconomic Status and Other Covariates

Family socioeconomic status was indexed using parental education and household income, following the approach described in the PISA 2009 Technical Report (2011). Parents reported the highest level of parental education and current household income. The two indicators were standardized within the sample and summed to produce a composite SES score, with higher scores indicating higher family socioeconomic status. SES was included as a covariate because differences in family educational and economic resources may be associated with children’s literacy-related experiences and early reading development (Cheung et al., 2021; Dulay et al., 2018).

Child age, gender, and nonverbal intelligence were included as covariates because they may be associated with EF task performance, early reading performance, or both. Age was included because both executive function and character reading develop rapidly during the preschool years. Nonverbal intelligence was included to account for broader individual differences in general cognitive ability that may be related to performance on both executive-function and reading tasks. Gender was included because previous research has shown gender-related differences in early literacy and executive-function performance (Deasley et al., 2018; Yamamoto & Imai-Matsumura, 2019). Age was calculated at T1 from the child’s date of birth and assessment date.

Kindergarten level (K1 and K2) was treated separately as a developmental grouping variable in the exploratory analyses examining whether associations between executive function and character reading differed across kindergarten levels.

### 2.3. Procedures

Data were collected at three occasions spanning approximately one year: T1: June–July 2023; T2: January 2024; T3: July 2024. At T1, children completed the single-character-reading task, the three EF tasks, and the nonverbal intelligence measure. Character reading was reassessed at T2 and T3. Parent-reported demographic and SES information was collected at T1.

Assessments were administered individually by trained examiners in a quiet room at the kindergarten. Examiners followed standardized administration and scoring procedures. Children were offered breaks when needed. The online nonverbal intelligence task was completed under direct examiner supervision.

### 2.4. Analytic Strategy

Analyses were conducted in R, using lavaan 0.6–21 (Rosseel, 2012). Preliminary analyses included descriptive statistics, zero-order correlations, observed change across adjacent assessments, and examination of ceiling concentration in character-reading scores. Models were estimated using robust maximum likelihood (MLR).

Change in character reading was examined using latent growth modeling. Because observed gains were larger from T1 to T2 than from T2 to T3 and an initial linear model produced an inadmissible negative residual variance at T3, the primary analysis used a latent-basis growth model (Grimm et al., 2017). Intercept loadings were fixed at 1 across occasions, whereas slope loadings were fixed at 0 at T1 and 1 at T3, with the T2 loading freely estimated. Thus, the intercept represented initial reading status, and the slope represented total latent change from T1 to T3. Reading residual variances were constrained to equality across occasions because freely estimated residuals produced an inadmissible solution.

Working memory, inhibitory control, and cognitive flexibility were entered simultaneously as correlates of the latent intercept and as prospective correlates of the latent slope. Age, gender, nonverbal intelligence, and socioeconomic status were included as covariates of both growth factors. Continuous predictors were grand-mean centered. Because EF and reading were both assessed at T1, associations with the intercept were interpreted as concurrent, whereas associations between baseline EF and the slope were interpreted as prospective associations with subsequent change. Model fit was evaluated using the robust *χ*^2^ statistic, CFI, TLI, RMSEA with 90% confidence intervals, and SRMR.

Exploratory follow-up analyses examined whether the two focal associations identified in the primary model differed between K1 and K2: working memory with initial reading status and cognitive flexibility with total reading change. K1 served as the reference group, and age was centered within kindergarten level because of its close association with K1/K2 status. Significant interactions were followed by simple-slope estimates within each kindergarten level. Given the unequal group sizes and the relatively small K1 subgroup, the moderation analyses were treated as exploratory. The smaller K1 sample reduced the statistical power and precision of the kindergarten-level interaction and simple-slope estimates; therefore, these results were interpreted cautiously and as preliminary evidence of possible developmental heterogeneity.

Missing data were handled primarily using FIML-X in lavaan, which retained cases with missing values on exogenous covariates and therefore used the full baseline sample of 131 children. Reading data were available for 131, 114, and 109 children at T1, T2, and T3, respectively; IQ and SES were available for 125 and 116 children. Under the missing-at-random assumption, FIML uses all available observed information to estimate model parameters (Enders, 2010). Attrition analyses compared children with and without T3 reading data on baseline characteristics, with detailed results reported in the Supplementary Materials.

Sensitivity analyses examined the robustness of the primary findings to alternative analytic decisions. The latent-basis model was re-estimated using ordinary FIML and complete cases; an alternative residual specification fixed the T1 and T3 reading residual variances to zero; and the primary and K1/K2 moderation models were re-estimated after applying an empirical-logit transformation to the bounded 0–27 reading scores. Finally, working memory and cognitive flexibility were examined in separate moderation models to assess whether the focal K1/K2 interactions depended on simultaneous adjustment for the other EF measures. Unless otherwise noted, sensitivity analyses used the same latent-basis specification, MLR estimator, and covariates as the primary model. Full results are reported in the Supplementary Materials.

## 3. Results

### 3.1. Descriptive Statistics and Zero-Order Correlations

**Figure 1:**
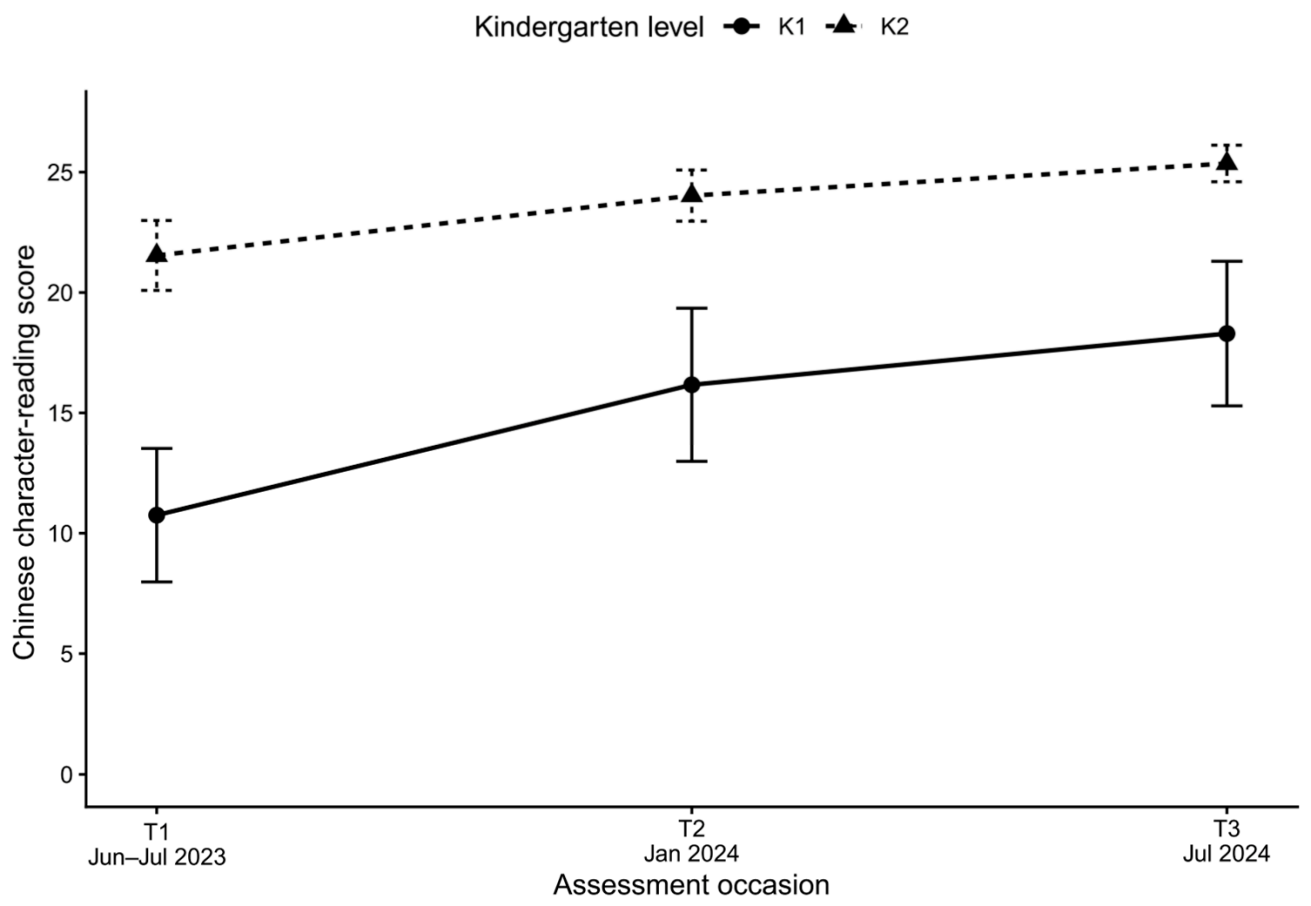
Observed reading trajectories.

Descriptive statistics for character reading are presented in Table 1. Mean character-reading scores increased from T1 (M = 17.92, SD = 9.19) to T2 (M = 21.54, SD = 7.48) and T3 (M = 23.16, SD = 6.40). The observed trajectories suggested greater gains between T1 and T2 than between T2 and T3 (Figure 1). K2 children had higher mean reading scores than K1 children at each assessment.

Character-reading distributions became increasingly negatively skewed over time, particularly among K2 children (Table 2). The proportion of children attaining the maximum score increased from 17.56% at T1 to 38.60% at T2 and 50.46% at T3. This upper-bound concentration was especially pronounced in K2, where the proportion attaining the maximum increased from 22.99% at T1 to 60.00% at T3; the corresponding proportions in K1 were 6.82% and 29.41%.

Zero-order correlations are presented in Table 3. Age was positively associated with all three EF measures and character reading at each assessment. Working memory showed moderate-to-strong positive correlations with character reading at T1, T2, and T3 (*r*s = 0.50–0.59). Cognitive flexibility was positively associated with T1 character reading (*r* = 0.36) but showed weak and nonsignificant correlations with T2 and T3 reading. Inhibitory control showed a small positive association with T1 reading (*r* = 0.19) but was not significantly related to reading at the later assessments. Nonverbal IQ was positively associated with reading at all three occasions, whereas gender was not significantly associated with reading. Character-reading scores were strongly correlated across assessments (*r*s = 0.80–0.92).

### 3.2. Primary Latent Growth Model

The primary analysis used raw character-reading scores, robust maximum likelihood estimation, FIML-X, and equal residual variances across the three reading assessments. The latent-basis model converged to a proper solution, with no negative residual variances or other inadmissible estimates. The model showed good fit: scaled *χ*^2^(9) = 11.03, *p* = .273, robust CFI = 0.991, robust TLI = 0.975, robust RMSEA = 0.063, 90% CI [0.000, 0.135], and SRMR = 0.016.

The freely estimated T2 slope loading was λ = 0.662, SE = 0.043, 95% CI [0.578, 0.746]. Because the slope loadings at T1 and T3 were fixed at 0 and 1, respectively, the T2 loading indicates that approximately 66% of the total latent change from T1 to T3 had occurred by T2. Thus, character-reading growth was decelerating, with greater improvement during the first interval than during the second, consistent with the descriptive pattern shown in Figure 1.

Table 4 presents the associations of the EF components and covariates with initial reading status and total reading change. Working memory was positively associated with initial character-reading status, b = 1.965, SE = 0.431, 95% CI [1.120, 2.809], β = 0.348, *p* < .001. No statistically significant association was detected between inhibitory-control task performance and initial status, b = 0.051, SE = 0.310, β = 0.013, *p* = .869. Cognitive flexibility showed a small positive coefficient at the conventional significance threshold, b = 0.664, SE = 0.339, 95% CI [−0.001, 1.330], β = 0.150, *p* = .050.

Among the covariates, age, b = 3.069, SE = 1.225, β = 0.211, *p* = .012, and nonverbal IQ, b = 0.141, SE = 0.068, β = 0.138, *p* = .037, were positively associated with initial reading status. Gender and socioeconomic status were not significant.

For total reading change, cognitive flexibility showed a significant negative association, b = −0.775, SE = 0.242, 95% CI [−1.249, −0.301], β = −0.330, *p* = .001. Working memory was not significantly associated with total change, b = −0.285, SE = 0.311, β = −0.095, *p* = .359, and no statistically significant association was detected between inhibitory-control task performance and total change, b = 0.099, SE = 0.196, β = 0.047, *p* = .612. None of the demographic covariates significantly predicted total change.

### 3.3. Moderation Analyses

Exploratory follow-up moderation analyses examined whether the two focal associations identified in the primary model differed between K1 and K2. Specifically, we tested whether kindergarten level moderated the association between working memory and initial reading status and the association between cognitive flexibility and total reading change. Age was centered within kindergarten level in these analyses. The moderation model converged to a proper solution and showed good fit: *χ*^2^(14) = 18.23, *p* = .196, robust CFI = 0.992, robust TLI = 0.982, robust RMSEA = 0.047, and SRMR = 0.014.

**Figure 2:**
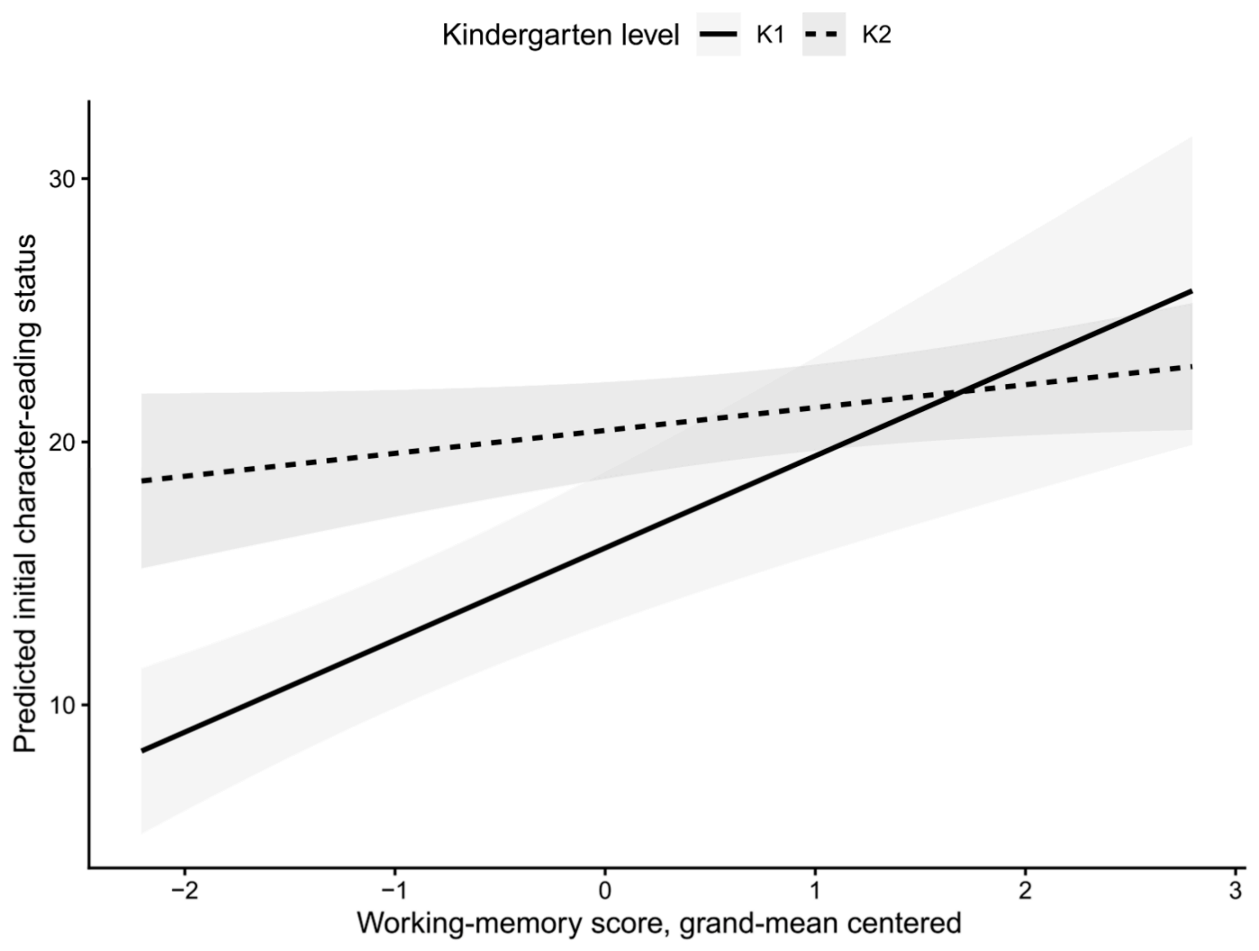
Working memory by kindergarten level.

Kindergarten level significantly moderated the association between working memory and initial reading status, b = −2.631, SE = 0.821, 95% CI [−4.239, −1.023], β = −0.302, *p* = .001. Simple-slope analyses showed that working memory was strongly and positively associated with initial status in K1, b = 3.501, SE = 0.720, 95% CI [2.090, 4.913], *p* < .001. The corresponding association was weaker and did not reach significance in the model for K2, b = 0.870, SE = 0.475, 95% CI [−0.062, 1.802], *p* = .067. This interaction is displayed in Figure 2. At the mean values of the other predictors, K2 children had higher initial reading status than K1 children, b = 4.470, SE = 1.677, 95% CI [1.184, 7.756], β = 0.231, *p* = .008.

Kindergarten level also moderated the association between cognitive flexibility and total reading change, b = 1.095, SE = 0.405, 95% CI [0.302, 1.889], β = 0.351, *p* = .007.

**Figure 3:**
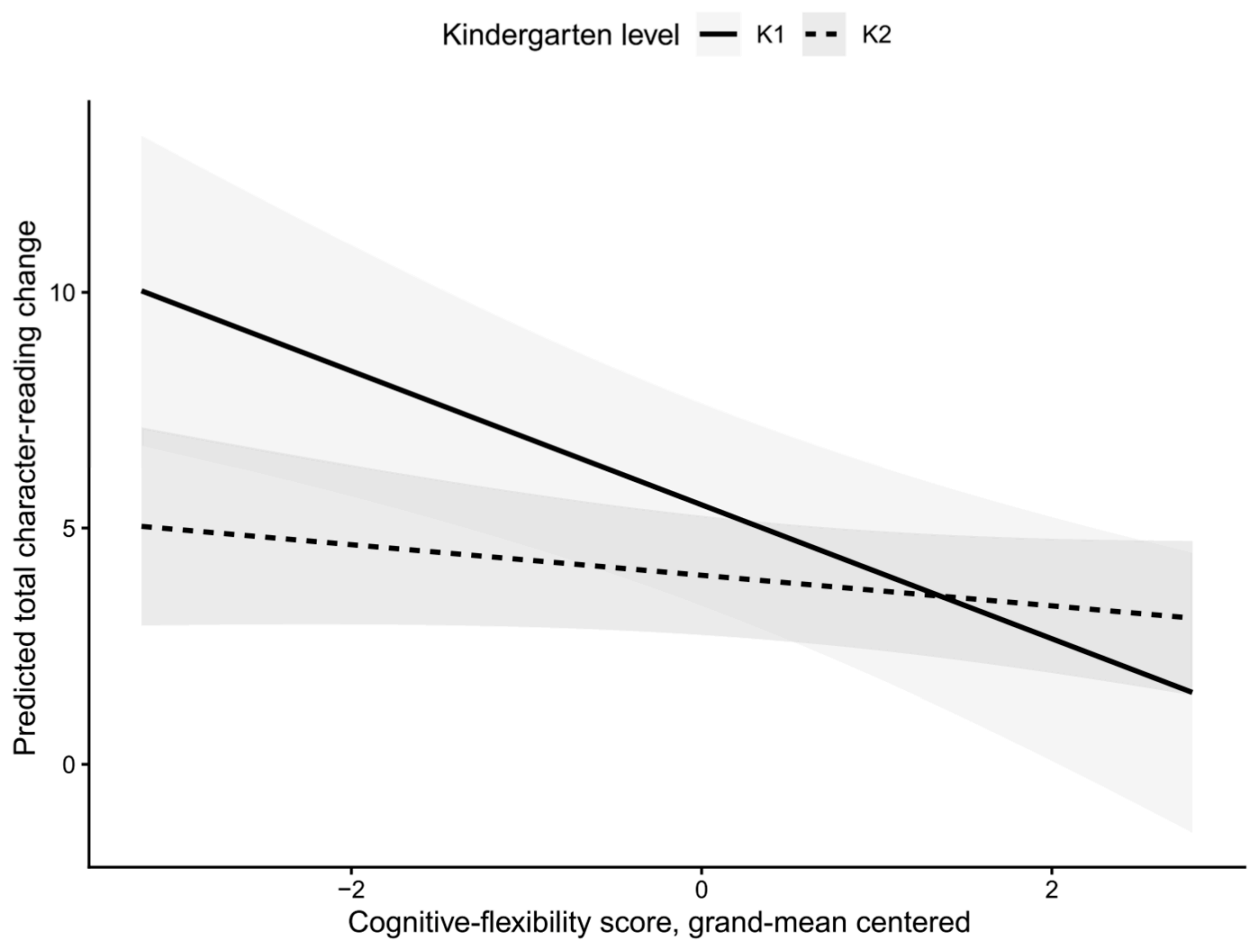
Cognitive flexibility by kindergarten level.

Cognitive flexibility was negatively associated with total change in K1, b = −1.420, SE = 0.386, 95% CI [−2.175, −0.664], *p* < .001. The association was smaller and nonsignificant in K2, b = −0.324, SE = 0.234, 95% CI [−0.783, 0.135], *p* = .166. Figure 3 illustrates this interaction. After accounting for the predictors, K1 and K2 did not differ significantly in total change, b = −1.493, SE = 1.210, β = −0.145, *p* = .217.

### 3.4. Sensitivity Analysis

Sensitivity analyses indicated that the principal findings were generally stable across alternative missing-data approaches, an alternative residual variance specification, and an empirical-logit transformation of the bounded reading scores. Working memory remained positively associated with initial reading status, cognitive flexibility remained negatively associated with total reading change, and inhibitory control remained unrelated to either growth factor. The kindergarten-level difference in the cognitive flexibility–change association was also relatively robust, whereas evidence that the working-memory association was stronger in K1 was less consistent across specifications (see Supplementary Materials).

## 4. Discussion

This study examined how baseline performance on working memory, inhibitory control, and cognitive-flexibility tasks was associated with initial status and subsequent change in Chinese character reading across three assessments in preschool children. The three executive-function measures showed distinct patterns of association with reading. Working memory was consistently and positively associated with initial character-reading status, whereas it was not associated with subsequent reading change. Inhibitory control showed no unique association with either initial status or change, although interpretation of this null finding is limited by the restricted variability in the inhibitory-control measure. Cognitive flexibility did not show a consistent association with initial reading status but was negatively associated with subsequent reading change. Although this negative association was reproduced across several sensitivity analyses, its interpretation should remain cautious given the modest reliability of the cognitive-flexibility measure, increasing ceiling compression in the reading outcome, and developmental heterogeneity across kindergarten levels. Overall, the findings suggest that baseline executive-function task performance was differentially associated with concurrent character-reading status and subsequent short-term change, but they do not support a simple developmental shift from reliance on working memory to reliance on cognitive flexibility.

### 4.1. Working Memory and Initial Character-Reading Status

Working-memory task performance showed the clearest and most consistent association with initial character-reading status. Children who performed better on the backward digit-span task also knew more Chinese characters at the first assessment, even after age, nonverbal intelligence, socioeconomic status, gender, and the other executive-function components were taken into account. This association was also stable across the sensitivity analyses. The finding therefore suggests a relatively specific role for working memory in individual differences in the character knowledge that children have accumulated during the preschool years. More broadly, this pattern is consistent with meta-analytic evidence showing reliable associations between working memory and reading across development (Peng et al., 2018), as well as studies specifically involving young Chinese readers (Yang & Qiao, 2021; Ren et al., 2022).

Baddeley’s multicomponent model (Baddeley, 2000) provides a useful framework for interpreting this association. Within this framework, working memory includes partly distinct systems for maintaining verbal and phonological information and visuospatial information, together with executive processes that support the active manipulation and coordination of information. The backward digit-span task used in the present study relies heavily on maintaining verbally coded information while also requiring its active reordering. It therefore primarily reflects verbal working-memory processes rather than the full range of working-memory functions. Such processes may be relevant to early character reading because learning an unfamiliar character requires children to maintain its pronunciation and link that pronunciation with the visually presented character form. Stronger verbal working-memory performance may therefore support the temporary maintenance and manipulation of pronunciation-related information while character–sound associations are being established and retrieved.

This interpretation is consistent with Yang and Qiao (2021), who found that working memory accounted for unique variance in character reading among Chinese kindergarten children and, when verbal and visuospatial working memory were considered simultaneously, verbal working memory showed the unique association with character reading. This finding is particularly relevant to the present study because the backward digit-span task also emphasized verbally coded information.

At the same time, Chinese character learning places substantial visuospatial demands. Characters are composed of strokes and components arranged within complex two-dimensional configurations, and children must encode and maintain these spatial relationships when forming orthographic representations. Within Baddeley’s framework, such processes would be expected to draw more strongly on visuospatial working memory. Because the present study did not include a visuospatial working-memory measure, it cannot determine the relative contributions of verbal and visuospatial working-memory processes to early character learning.

Converging evidence also comes from longitudinal research. Ren et al. (2022) found that executive-function abilities assessed during preschool were related to trajectories of Chinese word reading from preschool through Grade 3, with working memory particularly associated with children’s reading level during the preschool period. Evidence from children with early reading difficulties provides a complementary perspective. Fong and Ho (2022) found that Chinese kindergarten children identified as poor readers performed more weakly than normal readers across EF measures, and working memory was significantly related to word reading among the poor readers. Although their group-based design differs from the present study of typically developing children, these findings further indicate that working-memory differences are relevant to early Chinese reading performance. Together, the present and previous findings support the view that working memory is one cognitive resource associated with how much character knowledge children have acquired by the preschool period, rather than merely reflecting a general association between executive functioning and academic performance.

In contrast, backward digit-span performance was not uniquely associated with subsequent character-reading change over the one-year period. The distinction between initial status and change is theoretically informative. Initial status reflects children’s accumulated learning before and up to the first assessment, whereas the growth factor captures individual differences in how much additional character knowledge was acquired during the subsequent observation period. Verbal working memory may contribute importantly to the encoding, maintenance, and retrieval processes involved in building an initial repertoire of character representations, while variation in later gains may increasingly reflect a broader combination of language skills, print exposure, instructional experiences, and other reading-specific abilities. This interpretation is also compatible with evidence that the relation between working memory and reading varies with developmental level and reading demands rather than remaining constant across stages of reading acquisition (Peng et al., 2018). Importantly, the present pattern should not be interpreted as showing that working memory becomes unimportant as reading develops. Instead, it suggests that, under the conditions examined here, verbal working memory was more strongly related to existing individual differences in character knowledge than to individual differences in short-term subsequent growth during the period examined.

The modality and measurement of the working-memory measure should also be considered when interpreting this pattern. The backward digit-span task provides one indicator of working-memory performance and emphasizes the maintenance and manipulation of verbally coded information; it does not capture the full range of verbal and visuospatial working-memory processes that may contribute to Chinese literacy learning. Nor can a single task represent the broader working-memory construct. Nevertheless, the prominence of verbal working memory in the present results is consistent with Yang and Qiao’s (2021) finding that verbal, rather than visuospatial, working memory uniquely accounted for character reading when both were considered simultaneously. Future studies using multiple working-memory tasks could clarify whether different working-memory components contribute differently to the acquisition and growth of Chinese character knowledge.

### 4.2. Inhibitory Control and Early Character-Reading Development

Inhibitory-control task performance showed a different pattern from working memory. Although inhibitory control was modestly correlated with character reading at T1, no statistically significant unique association was detected between inhibitory-control task performance and either initial reading status or subsequent change once working memory, cognitive flexibility, and the covariates were considered. However, this null finding should be interpreted cautiously. Scores on the Grass-Snow task were highly concentrated near the maximum, resulting in substantial range restriction and limited sensitivity to individual differences in inhibitory control. This restricted variability may have attenuated associations with character reading and limits the extent to which the present study can evaluate the role of inhibitory-control reliably.

The absence of a significant unique association is nevertheless broadly consistent with some previous findings. For example, in a one-year study of 523 Hong Kong kindergarteners, Fung et al. (2020) found that baseline inhibitory control, attention shifting, and working memory did not prospectively predict later Chinese word reading during the kindergarten period, although working memory was the strongest concurrent EF correlate of word reading. More broadly, meta-analytic and longitudinal evidence suggests that relations between inhibition and reading vary across developmental periods, reading outcomes, and the extent to which other executive-function components are considered simultaneously (Spiegel et al., 2021; T. Zhou et al., 2026; Zhao et al., 2024). Related work has likewise suggested that EF–reading relations may change as children move from kindergarten into formal schooling (Liu et al., 2019; Ren et al., 2022). Thus, the present finding should be viewed as one instance within a heterogeneous literature rather than as evidence that inhibitory control is unimportant for early reading.

There are also theoretical reasons to expect inhibitory control to be relevant to character reading under some conditions. From a lexical-processing perspective, recognition of an isolated character may activate interconnected orthographic and phonological representations, including competing representations when different characters share visual features or pronunciations (Perfetti et al., 2005). In such situations, inhibitory control may contribute to resolving competition and maintaining the appropriate response. The extent to which this process depends on domain-general inhibitory control, however, may vary with children’s reading knowledge, the characteristics of the reading task, and the sensitivity of the inhibitory-control measure. Evidence from other reading contexts similarly suggests that inhibition may be more detectable when reading places stronger demands on resolving competition or suppressing inappropriate responses (e.g., Qiu et al., 2023).

Accordingly, the present results are best interpreted as showing that the inhibitory-control performance captured by the Grass-Snow task did not account for detectable unique variance in initial character-reading status or subsequent change in this sample. Given the pronounced ceiling effect, this should not be taken as evidence against a functional role of inhibitory control in early character reading. Future studies should use inhibitory-control tasks with greater difficulty and discriminative sensitivity for preschool children and should examine whether associations vary across reading tasks that differ in competition and control demands.

### 4.3. Cognitive Flexibility and Change in Character Reading

Cognitive-flexibility task performance showed a different and less straightforward pattern from that of working memory. It was not consistently associated with children’s initial character-reading status, but higher baseline cognitive-flexibility performance was associated with smaller subsequent increases in character reading. This negative coefficient does not indicate that children with stronger cognitive flexibility lost reading skills or that cognitive flexibility was detrimental to reading. Character-reading scores increased on average over time; rather, the coefficient indicates that children with higher cognitive-flexibility scores tended to show less additional measured growth during the observation period. The negative association was also reproduced across alternative missing-data approaches, the alternative residual-variance specification, and the empirical-logit analysis. Thus, although the direction was unexpected, it was not specific to a single analytic specification.

The negative direction contrasts with the generally positive role proposed for cognitive flexibility in reading. Cognitive flexibility may support readers in coordinating different sources of information and shifting among orthographic, phonological, semantic, and contextual representations as task demands change. Earlier work with beginning readers found positive associations between cognitive flexibility and word reading or reading comprehension (Colé et al., 2014), and recent evidence likewise links stronger cognitive flexibility with emergent literacy among kindergarten children (Tal & Shaul, 2024). In Chinese, Fong and Ho (2022) found that cognitive flexibility predicted reading outcomes among typically developing kindergarten readers, while Ren et al. (2022) reported that parent-rated cognitive flexibility was positively related to growth in Chinese word reading from preschool through the early primary grades. Among older Chinese children, attention shifting has also been associated with word reading fluency (H. Zhou & Jiang, 2024). Therefore, the present negative association should not be interpreted as evidence for a general negative role of cognitive flexibility in literacy development.

One possible explanation concerns the developmental demands of the cognitive-flexibility task itself. Performance on DCCS-type tasks does not reflect switching ability alone. Successful performance also requires children to maintain the currently relevant rule, suppress interference from the previously relevant rule, and shift efficiently when the rule changes. These component demands continue to develop substantially during early childhood, and switching can carry measurable costs even when children are able to apply the new rule successfully (Blackwell & Munakata, 2014; Chevalier et al., 2015). Accordingly, higher performance on a DCCS-type task may reflect a particular combination of rule maintenance, switching, and interference control rather than a unitary level of “flexibility”.

The distribution of character-reading performance may also have contributed to the observed association. Children with stronger cognitive-flexibility performance tended to have stronger reading performance at the beginning of this study, while character-reading scores became increasingly compressed near the upper limit over time. Children who entered this study with more established character knowledge consequently had less observable room for additional gains on the 27-item measure. The negative association between cognitive flexibility and reading change remained when an empirical-logit transformation was applied, but this transformation does not restore variability lost when children reach the maximum score. Therefore, ceiling effects, particularly at T3, may distort the estimation of the latent growth factor by making part of the observed slope reflect the approach to the test ceiling rather than unrestricted change in underlying reading ability. Consequently, the negative association should be interpreted with caution, and the empirical-logit sensitivity analysis should be viewed as evidence that the finding was not specific to the raw-score metric rather than as a solution to the censoring problem.

The exploratory kindergarten-level findings also suggest that developmental heterogeneity may be relevant. The association appeared stronger in K1 than in K2, but these subgroup analyses were based on small and unequal samples and should be interpreted cautiously. They do not establish distinct developmental stages or demonstrate that the role of cognitive flexibility changes systematically from K1 to K2. Rather, they indicate that the association may not be uniform across the preschool period and warrants examination in larger longitudinal samples.

The result nevertheless warrants caution for measurement reasons. Cognitive flexibility was assessed with a single task, and its internal consistency was modest. Performance-based EF tasks necessarily reflect both the intended executive process and task-specific demands, making conclusions about an underlying EF component less secure when only one task is available (Friedman & Miyake, 2017; Karr et al., 2018). In addition, with only three reading assessments, the latent-basis model provides a parsimonious description of nonlinear change but cannot characterize more complex developmental trajectories.

Taken together, the findings complicate a simple account in which stronger executive functioning uniformly promotes faster early reading growth. Higher baseline cognitive-flexibility performance was associated with less measured character-reading change, but the meaning of this association remains uncertain. The pattern may reflect developmental differences in the balance between rule maintenance, switching, and interference control, characteristics of the reading measure, or other unmeasured features of early character learning. Future longitudinal studies should use multiple indicators of cognitive flexibility, directly assess switching costs and interference control, include reading measures with a wider difficulty range, and repeatedly assess both executive functions and reading. Such designs would provide a stronger basis for determining whether the present negative association reflects a replicable developmental pattern or task-specific characteristics of the measures used here.

### 4.4. Differences Between K1 and K2

The exploratory moderation analyses suggested that the associations between baseline executive-function task performance and character reading were not entirely uniform across the two kindergarten levels. In the simultaneous model, the positive association between working memory and initial character-reading status was stronger in K1 than in K2. Cognitive flexibility also showed a kindergarten-level difference, with the negative association with subsequent reading change being stronger in K1 than in K2. These findings suggest that developmental context may condition how individual differences in executive-function task performance are related to early character reading. However, because the subgroup analyses were based on relatively small and unequal samples, these patterns should be considered exploratory.

The working-memory pattern requires particular qualification. Although the Working Memory × Kindergarten Level interaction was significant when all three executive-function components were considered simultaneously, the interaction was attenuated when working memory was examined separately. In that model, working memory was positively associated with initial reading status in both K1 and K2, although the association was numerically stronger in K1. The transformed-score sensitivity analysis similarly showed significant positive simple slopes in both groups. Thus, the findings do not indicate that working memory is relevant only to younger preschoolers. Rather, they provide preliminary evidence that the association between verbal working-memory task performance and existing character knowledge may be stronger earlier in the preschool period. This pattern is compatible with longitudinal Chinese research showing that working memory is particularly related to children’s level of word reading during the preschool period (Ren et al., 2022), but the present moderation findings require replication in larger samples.

The kindergarten-level difference involving cognitive flexibility was more consistent across model specifications. The negative association between cognitive flexibility and reading change was stronger in K1 than in K2, and this interaction remained evident in the empirical-logit and cognitive-flexibility-only analyses. However, this pattern should be interpreted cautiously. As discussed above, ceiling compression in the reading measure limits the interpretation of latent change, and the empirical-logit transformation does not restore variability lost at the upper bound. The K1/K2 pattern cannot establish that the negative association reflects a distinct developmental process. Nor should it be interpreted as showing that cognitive flexibility is detrimental to reading among younger children. Previous studies have generally found positive relations between cognitive flexibility or broader executive functioning and Chinese reading outcomes (Fong & Ho, 2022; Liu et al., 2019; Xu et al., 2024).

The Dual Mechanisms of Control framework provides one possible account of why associations between executive function and reading may vary across this developmental period (Braver, 2012). This framework distinguishes reactive control, which is recruited in response to immediate demands, from proactive control, which involves the sustained maintenance of goal-relevant information in anticipation of upcoming demands. Across early childhood, children become increasingly able to maintain task goals proactively rather than relying primarily on control triggered by immediate events. Applied cautiously to the present findings, such developmental changes in control may alter how working-memory and cognitive-flexibility processes are recruited during character learning. Younger children may depend more heavily on the immediate maintenance of task-relevant verbal information while character-sound associations are still being established, whereas older preschoolers may increasingly draw on more sustained and coordinated forms of control as their character knowledge becomes more established. However, the present study did not directly assess reactive or proactive control, and K1 and K2 should not be equated with these two control modes. The DMC framework therefore offers a possible interpretation of the observed moderation rather than a mechanism demonstrated by the current data.

A broader reading-development perspective leads to a similar conclusion. The Active View of Reading emphasizes that reading develops through interactions among multiple language, cognitive, and self-regulatory processes whose contributions may vary with children’s knowledge and the demands of the reading task (Duke & Cartwright, 2021). From this perspective, the relation of a domain-general executive-function skill to reading need not remain constant across development. K1 and K2 children differed not only in age but also in their starting levels of character knowledge and likely in their cumulative literacy experiences. The same executive-function performance may therefore be associated differently with reading depending on the degree to which character representations have already been established and on the cognitive demands of subsequent learning. Previous longitudinal studies likewise suggest that relations between executive functioning and Chinese word reading change across development (Liu et al., 2019; Ren et al., 2022), although these studies do not directly test the specific mechanisms proposed here.

Therefore, the present findings do not support a developmental transfer or handoff from working memory to cognitive flexibility. Such an interpretation would require evidence that the contribution of working memory decreases while that of cognitive flexibility increases within the same children as they develop. Executive functions were assessed only at baseline in the present study, and K1 and K2 represented different groups of children rather than successive developmental stages observed within the same individuals. Moreover, cognitive flexibility did not show a stronger positive contribution among the K2 children. The moderation findings are better viewed as preliminary evidence that associations between executive function and reading may vary across the preschool period. The DMC and Active View frameworks provide plausible ways to conceptualize this variation, but direct tests of the underlying developmental mechanisms will require repeated assessment of executive functions and reading within larger longitudinal samples.

### 4.5. Limitations and Future Directions

Several limitations should be considered when interpreting the findings. First, each executive-function component was assessed with a single task. Performance-based EF measures capture the targeted executive process together with task-specific demands, making construct-level interpretation less secure when only one indicator is available (Miyake et al., 2000; Friedman & Miyake, 2017). In addition, the inhibitory-control task showed ceiling effects. This restricted variability likely reduced the task’s sensitivity to individual differences and limited the ability of the present study to evaluate associations between inhibitory control and character reading. Future studies should use multiple indicators of each EF component, including both verbal and visuospatial working-memory tasks, and use inhibitory-control measures with a wider difficulty range and greater discriminative sensitivity for preschool children.

Second, the reading outcome was a brief single-character-naming task. Although appropriate for assessing emergent character knowledge, it does not capture broader literacy skills such as multi-character word reading, fluency, or comprehension. Scores also became increasingly compressed near the upper limit, particularly at T3 and among K2 children. This ceiling compression reduces observable individual differences at higher levels of performance and may distort estimation of the latent growth factor because observed change can partly reflect an approach to the test maximum rather than unrestricted change in underlying reading ability. Although the main findings were similar when an empirical-logit transformation was applied, such transformations cannot recover latent variability that has already been lost because of censoring. Future studies should use reading measures with a wider difficulty range and, where substantial censoring remains, consider statistical approaches designed for bounded or censored longitudinal outcomes, such as censored growth models or longitudinal models based on item response theory.

Third, executive functions were measured only at baseline, whereas reading was assessed repeatedly across three occasions. The present design therefore addresses how baseline EF task performance relates to initial reading status and subsequent change, but it cannot examine how EF and reading develop together or influence one another over time. This is important given evidence for reciprocal longitudinal relations between executive functioning and Chinese word reading (Liu et al., 2019). Additional waves and repeated assessment of both EF and reading would allow stronger tests of developmental coupling and more complex growth trajectories.

Fourth, the kindergarten-level moderation analyses were limited by the relatively small and unequal subgroup sizes. The K1 group included 44 children at baseline, compared with 87 in K2, and the number of K1 children with reading data decreased further across measurement occasions. These features reduced the statistical power and precision of the interaction and simple-slope estimates, particularly for K1. Accordingly, the K1/K2 findings should be regarded as exploratory and preliminary rather than as definitive evidence of developmental differences in EF–reading associations. Replication with larger and more balanced samples is needed.

Finally, the models did not include several language, literacy, and environmental factors that may also contribute to character-reading development, such as morphological awareness, phonological awareness, print exposure, and home literacy experiences. Attrition was also related to some baseline characteristics, although the principal findings were similar across FIML-X, ordinary FIML, and complete-case analyses. In addition, participants came from a single kindergarten, limiting generalizability to other educational contexts and populations. Future multi-site longitudinal studies following children into primary school and incorporating richer measures of EF, language, literacy, and learning environments would help clarify the conditions under which different executive-function components are associated with Chinese reading development.

## 5. Conclusions

The present study suggests that executive-function task performance is differentially associated with early Chinese character reading. Performance on the verbal working-memory task showed the clearest and most consistent relation with initial character-reading status, suggesting that verbally mediated working-memory processes are particularly relevant to the character knowledge children have accumulated by the preschool period. In contrast, cognitive flexibility showed an unexpected negative association with subsequent measured change, a pattern that was reproduced across several sensitivity analyses and points to a more complex developmental relation between cognitive flexibility and early character learning. No statistically significant association was detected for inhibitory-control task performance, although the pronounced ceiling effect in the Grass–Snow task limits the interpretation of this finding.

The exploratory K1/K2 analyses further suggested that these EF–reading associations were more pronounced among the younger K1 children. The relation between working-memory task performance and initial reading status was stronger in K1 in the simultaneous model, and the negative association between cognitive-flexibility task performance and subsequent change was also concentrated more strongly in K1. Together, these findings suggest that the contribution of domain-general executive processes to early character reading may be especially salient during the earlier preschool period, when character knowledge is less established and children are still building stable character–sound representations. This developmental pattern is consistent with the broader view that the cognitive processes supporting reading are not fixed, but change as children’s literacy knowledge and control processes develop.

Educationally, the findings highlight the importance of recognizing that early character reading is supported not only by language- and literacy-specific skills but also by broader cognitive processes. In particular, the consistent association between verbal working-memory task performance and initial character knowledge suggests that young children differ in the cognitive resources they can draw on while acquiring new characters. These findings do not by themselves identify a specific instructional method, but they provide a basis for future intervention research examining whether instructional approaches that take children’s cognitive demands into account can support early character learning. More broadly, this study shows the value of examining executive-function components separately and distinguishing their relations with existing reading knowledge from their relations with subsequent growth.

Future longitudinal studies using broader EF measures, more sensitive reading assessments, and repeated measurement across the transition to primary school will be important for testing the developmental mechanisms suggested by the present findings and for determining their implications for early literacy instruction.

## Figures and Tables

**Table 1:** Descriptive statistics for all variables.

Variable	N	Mean	SD	Median	Min	Max	Skewness	Kurtosis
Age	131	4.98	0.62	5.14	3.66	5.81	−0.65	−0.8
Gender	131	0.47	0.5	0	0	1	0.11	−2
Nonverbal IQ	125	89.9	8.83	89	77	123	0.8	0.78
SES	116	12.55	2.42	13	5	17	−0.73	0.12
Working memory	131	2.21	1.6	2	0	6	0.23	−0.44
Inhibitory control	131	14.84	2.26	16	7	16	−2.23	3.92
Cognitive flexibility	131	14.2	2.05	15	8	17	−0.64	0.31
Character reading T1	131	17.92	9.19	22	0	27	−0.69	−1.03
Character reading T2	114	21.54	7.48	26	0	27	−1.25	0.38
Character reading T3	109	23.16	6.4	27	0	27	−1.88	2.86

**Table 2:** Character-reading descriptives.

Wave	Group	N	Mean	SD	Median	Minimum	Maximum	Ceiling_n	Ceiling_Percent
T1	Total sample	131	17.92	9.19	22.0	0	27	23	17.56
T2	Total sample	114	21.54	7.48	26.0	0	27	44	38.60
T3	Total sample	109	23.16	6.40	27.0	0	27	55	50.46
T1	K1	44	10.75	9.12	10.0	0	27	3	6.82
T2	K1	36	16.17	9.40	15.5	0	27	10	27.78
T3	K1	34	18.29	8.61	21.5	0	27	10	29.41
T1	K2	87	21.54	6.81	25.0	1	27	20	22.99
T2	K2	78	24.03	4.71	26.0	10	27	34	43.59
T3	K2	75	25.36	3.30	27.0	10	27	45	60.00

**Table 3:** Zero-order correlations among variables.

Variable	1	2	3	4	5	6	7	8	9	10
1. Age	—									
2. Gender	−0.24 **	—								
3. Nonverbal IQ	0.27 **	−0.06	—							
4. SES	0.00	0.04	0.11	—						
5. Working memory	0.61 ***	−0.21 *	0.39 ***	0.14	—					
6. Inhibitory control	0.29 ***	−0.04	0.13	−0.09	0.20 *	—				
7. Cognitive flexibility	0.32 ***	−0.05	0.09	0.17	0.30 ***	0.22 *	—			
8. Character reading T1	0.52 ***	−0.15	0.35 ***	0.18 *	0.59 ***	0.19 *	0.36 ***	—		
9. Character reading T2	0.37 ***	−0.08	0.30 **	0.14	0.50 ***	0.11	0.12	0.88 ***	—	
10. Character reading T3	0.39 ***	−0.06	0.29 **	0.13	0.52 ***	0.12	0.12	0.80 ***	0.92 ***	—

Note. * *p* < 0.05; ** *p* < 0.01; *** *p* < 0.001.

**Table 4:** Primary latent-basis growth model.

Outcome	Predictor	b	SE	95% CI	Beta	*p*
Initial status	Working memory	1.965	0.431	[1.120, 2.809]	0.348	<.001
Initial status	Inhibitory control	0.051	0.310	[−0.556, 0.659]	0.013	.869
Initial status	Cognitive flexibility	0.664	0.339	[−0.001, 1.330]	0.150	.050
Initial status	Gender	−0.223	1.204	[−2.583, 2.137]	−0.012	.853
Initial status	Age	3.069	1.225	[0.668, 5.470]	0.211	.012
Initial status	Nonverbal IQ	0.141	0.068	[0.008, 0.273]	0.138	.037
Initial status	SES	0.393	0.287	[−0.170, 0.955]	0.105	.171
Total change	Working memory	−0.285	0.311	[−0.893, 0.324]	−0.095	.359
Total change	Inhibitory control	0.099	0.196	[−0.285, 0.484]	0.047	.612
Total change	Cognitive flexibility	−0.775	0.242	[−1.249, −0.301]	−0.330	.001
Total change	Gender	1.031	0.895	[−0.723, 2.784]	0.107	.249
Total change	Age	−1.424	0.931	[−3.247, 0.400]	−0.184	.126
Total change	Nonverbal IQ	−0.055	0.052	[−0.158, 0.047]	−0.102	.289
Total change	SES	−0.191	0.183	[−0.550, 0.167]	−0.097	.296

## Data Availability

The raw data supporting the conclusions of this article will be made available by the authors on request.

## Supplementary Materials

The following supporting information can be downloaded at: https://www.mdpi.com/article/10.3390/jintelligence14100266/s1, Figure S1: Sensitivity forest plot.
