# Supplementary material for: Differential Associations of Executive-Function Components with Initial Status and Change in Chinese Character Reading During Preschool
Source: J Intell. 2026 Oct 16;14(10):266. doi: 10.3390/jintelligence14100266 (PMC13705745; doi:10.3390/jintelligence14100266)

Sensitivity Analyses

Missing-data handling

The primary findings were consistent across the FIML-X analysis, ordinary FIML analysis, and complete-case analysis. All three models converged, produced admissible solutions, and showed adequate fit. The working-memory association with initial status was highly stable, with standardized coefficients ranging from $\beta = .325$ to $.348$; all corresponding p values were below .001. The negative association between cognitive flexibility and total change was also consistent, ranging from $\beta = -.249$ to $-.330$, with p values between .025 and .001.

In contrast, the association between cognitive flexibility and initial status was not stable across missing-data approaches. It was at the significance threshold in the FIML-X model but nonsignificant under ordinary FIML and complete-case analysis. Working memory and inhibitory control remained unrelated to total change, and inhibitory control remained unrelated to initial status.

Residual-variance constraints

A model fixing only the T3 residual variance to zero remained inadmissible and was not interpreted. Both the equal-residual model and the model fixing the T1 and T3 residual variances to zero were admissible. The equal-residual specification was retained as the primary model because it did not require the stronger assumption of zero occasion-specific residual variance at both endpoints. The central working-memory and cognitive-flexibility findings were similar under the endpoint-zero specification.

Empirical-logit transformation

To examine the influence of the bounded 0-27 reading scale, the models were repeated using empirical-logit-transformed reading scores. The transformed-outcome model converged,

was admissible, and showed adequate fit, $\chi^2(9) = 13.63$, $p = .136$, robust CFI = .986, robust TLI = .961, robust RMSEA = .076, and SRMR = .018. The latent-basis loading was $\lambda = .700$, 95% CI [.591, .809], again indicating decelerating growth.

The main associations were unchanged. Working memory remained positively associated with initial status, $\beta = .367$, $p < .001$, whereas cognitive flexibility remained negatively associated with total change, $\beta = -.349$, $p = .006$. Inhibitory control was unrelated to either growth factor.

The kindergarten-level interactions also remained significant after transformation. Kindergarten level moderated the working-memory association with initial status, $b = -0.554$, SE = 0.199, $\beta = -.270$, $p = .005$. On the transformed scale, working memory was positively associated with initial status in both K1, $b = 0.816$, $p < .001$, and K2, $b = 0.263$, $p = .025$, although the association was stronger in K1. Kindergarten level also moderated the cognitive-flexibility association with total change, $b = 0.211$, SE = 0.101, $\beta = .396$, $p = .036$. Cognitive flexibility was negatively associated with change in K1, $b = -0.243$, $p = .008$, but not in K2, $b = -0.032$, $p = .555$.

Separate EF models

The cognitive-flexibility moderation effect remained significant when cognitive flexibility was entered without the other EF components, $b = 1.213$, SE = 0.435, $\beta = .384$, $p = .005$. This indicates that the K1/K2 difference in the cognitive-flexibility association was not dependent on adjustment for working memory and inhibitory control.

By contrast, the working-memory-by-kindergarten interaction was reduced when working memory was entered without the other EF measures, $b = -1.698$, SE = 0.926, $\beta = -.198$, $p = .067$. Thus, although working memory was positively associated with initial reading in both

kindergarten levels, evidence that this association was stronger in K1 was less consistent across the simultaneous and separate-EF specifications. Figure S1 summarizes the stability of the two main EF associations across the sensitivity analyses.

Figure S1 Sensitivity forest plot

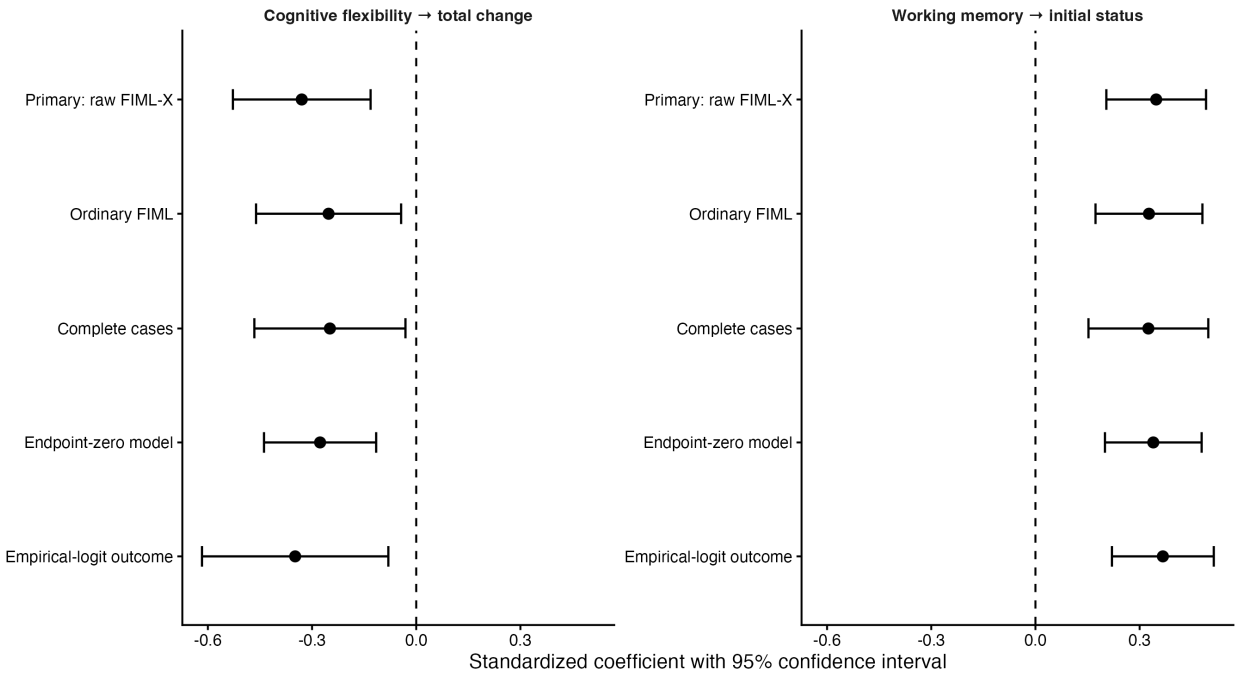

Supplement: Supplementary file 1 [file jintelligence-14-00266-s001.zip › jintelligence-4561932-supplementary.pdf]
